# Supplementary material for: Paper-Based ZnS:Cu Alternating Current Electroluminescent Devices for Current Humidity Sensors with High–Linearity and Flexibility
Source: Sensors (Basel). 2019 Oct 23;19(21):4607. doi: 10.3390/s19214607 (PMC6864801; doi:10.3390/s19214607)
Supplement: Supplementary file 1 [file sensors-19-04607-s001.pdf]

sensors

MDPI

Supplementary Materials

Paper-Based ZnS:Cu Alternating Current Electroluminescent Devices for Current Humidity Sensors with High–Linearity and Flexibility

Yaqin He, Mengyao Zhang, Nan Zhang, Danrong Zhu, Chun Huang, Ling Kang, Xiaofeng Zhou, Menghan Hu and Jian Zhang *

Shanghai Key Laboratory of Multidimensional Information Processing, East China Normal University, 500 Dongchuan Road, Shanghai 200241, China; 51171213022@stu.ecnu.edu.cn (Y.H.); 51181213046@stu.ecnu.edu.cn (M.Z.); 52171213016@stu.ecnu.edu.cn (N.Z.); 51171213080@stu.ecnu.edu.cn (D.Z.); 52151213018@stu.ecnu.edu.cn (C.H.); 52171213015@stu.ecnu.edu.cn (L.K.); xfzhou@ee.ecnu.edu.cn (X.Z.); mhhu@ce.ecnu.edu.cn (M.H.)

* Correspondence: jzhang@ee.ecnu.edu.cn

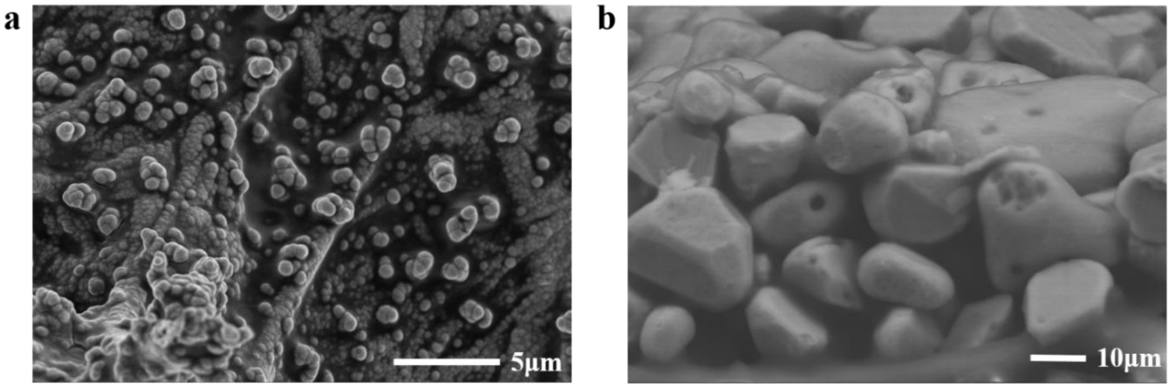

Figure S1. The SEM images of (**a**) Ni layer (**b**) ZnS:Cu.

Figure S1a shows the surface of Ni-P layer via electroless plating which can be found that lots of homogeneously dispersed Ni nanoclusters were grown on filter paper successfully. And from Figure S1b, it can be seen that ZnS:Cu particles wrapped by PB glue were tightly packed together.

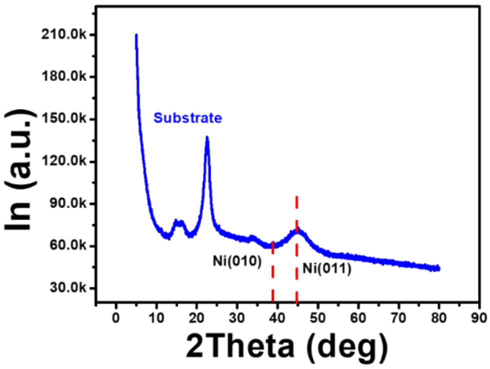

Figure S2. XRD spectrum of the Ni layer.

Figure S2 shows the XRD results. The characteristic peak of Ni ~44.8° and ~38.8° are visible, indicating that the Ni layer was successfully deposited.

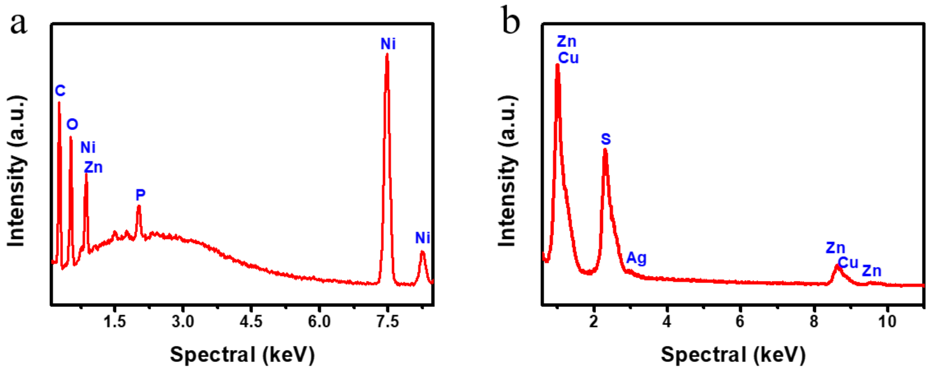

Figure S3. The EDS spectrum of (**a**) paper-based ACEL device and (**b**) phosphor layer.
